# MYOSLID: A Critical Modulator of Cancer Hallmarks

**DOI:** 10.3390/genes16030341

**Published:** 2025-03-14

**Authors:** Kanupriya Medhi, Sagarika Mukherjee, Aastha Dagar, Ashutosh Kumar Tiwari, Sia Daffara, Sanjana Bana, Vivek Uttam, Md Rizwan Ansari, Vikas Yadav, Hardeep Singh Tuli, Aklank Jain

**Affiliations:** 1 Non-Coding RNA and Cancer Biology Lab, Department of Zoology, Central University of Punjab, Ghudda, Bathinda 151401, Punjab, India; priyamedhi14@gmail.com (K.M.); sagarikamukherjee444@gmail.com (S.M.); aasthadagar2000@gmail.com (A.D.); ashutoshlokesh@gmail.com (A.K.T.); siadaffara@gmail.com (S.D.); sanjanabana@gmail.com (S.B.); vivekuttam52@gmail.com (V.U.); 2 3rd Floor, Jyoti Pinnacle Building, Survey No.11, Kondapur Village, Serilingampally Mandal, Ranga Reddy District, Hyderabad 500081, Telangana, India; rizwanindia@gmail.com; 3 Department of Translational Medicine, Clinical Research Centre, Skåne University Hospital, Lund University, 20213 Malmö, Sweden; vikas.yadav@med.lu.se; 4 Department of Bio-Sciences and Technology, Maharishi Markandeshwar (Deemed to be University), Mullana, Ambala 133207, India

**Keywords:** LncRNA, *MYOSLID*, cancer hallmarks, sustaining proliferative signaling, avoiding immune destruction and cell death resistance, activating invasion and metastasis, deregulating cellular metabolism, head and neck squamous cell carcinoma, colorectal cancer, osteosarcoma, gastric cancer, oral cell squamous carcinoma

## Abstract

Despite being the leading cause of death worldwide, cancer still lacks precise biomarkers for effective targeting, limiting efforts to reduce mortality rates. This review explores the role and clinical significance of a newly identified long non-coding RNA, *MYOSLID*, in cancer progression. *MYOSLID* has emerged as a critical modulator in cancer progression by influencing key hallmarks such as proliferation, immune evasion, metastasis, and metabolic reprogramming. It promotes tumor cell growth by stabilizing hypoxia-inducible factor 1 and acting as a competing endogenous RNA (ceRNA) to sequester tumor-suppressive microRNAs like miR-29c-3p, thereby enhancing oncogene expression. It facilitates immune evasion by upregulating PD-L1, suppressing T cell activation, and modulating necroptosis pathways involving RIPK1 and RIPK3. Additionally, *MYOSLID* drives metastasis by regulating epithelial–mesenchymal transition markers such as LAMB3 and Slug while promoting RAB13-mediated cytoskeletal remodeling and enhancing cancer cell invasion. We have obtained the expression of *MYOSLID* from TCGA and the ENCORI database. The expression of colorectal adenocarcinoma (COAD) and head and neck squamous cell carcinoma (HNSCC) is associated with poor prognosis and lower survival rate. Given its significant potential as a diagnostic biomarker and therapeutic target, further research is required to elucidate its precise molecular mechanisms and therapeutic applications in cancer treatment.

## 1. Introduction

Despite the advancement of modern technologies, cancer continues to rank as the second leading cause of death worldwide. A significant contributing factor to the high cancer mortality rate is the lack of specific and sensitive molecular markers that provide accurate diagnostic and prognostic information. As a result, extensive research is underway to identify reliable molecular markers, with particular emphasis on non-coding RNA-based markers [1]. The significance of miRNAs was highlighted by the fact that, recently, scientists received a Nobel Prize for their research in this area. We and other researchers have identified long non-coding RNA (lncRNA)-based molecular markers as critical contributors to the development of various cancers [2,3,4,5].

LncRNAs represent a diverse group of RNA molecules, typically exceeding 200 nucleotides in length, that do not code for proteins [6]. LncRNAs act as competing endogenous RNAs (ceRNAs) and have significant potential as diagnostic and prognostic biomarkers for various cancers.

Several lncRNAs such as *H19*, *DANCR* (differentiation antagonizing non-protein-coding RNA), *UCA1* (Urothelial Carcinoma Associated 1), *SFTA1P* (Surfactant Associated 1, Pseudogene), and *LUCAT1* function as an oncogene. At the same time, many lncRNAs act as tumor suppressors, such as *GAS5* (growth arrest-specific 5) and *BC069792* in breast cancer. LncRNA expression can affect clinicopathological features such as lymph node metastasis, age, gender, Ki-67 levels, tumor node metastasis stage, and histological grade [6,7,8,9,10,11].

**Figure 1:**
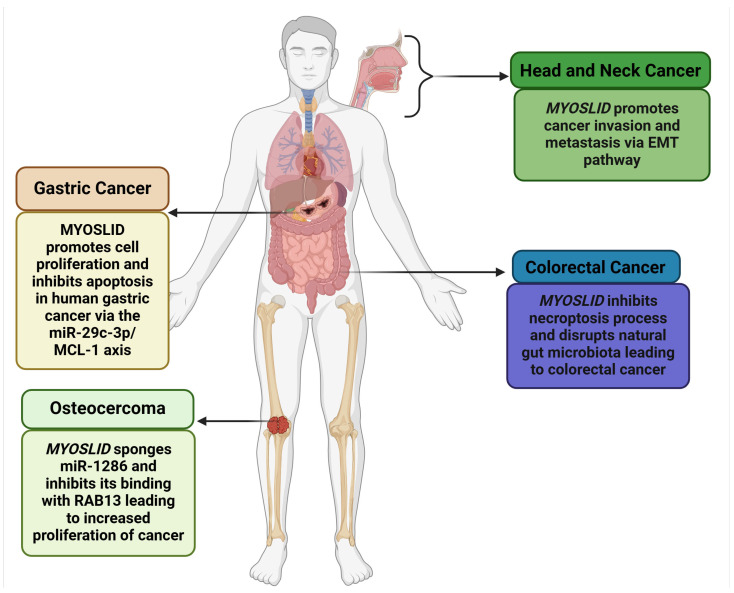
Role of *MYOSLID* in various cancers (Created with BioRender.com).

*MYOSLID* (Myocardin-Induced Smooth Muscle LncRNA Inducer of Differentiation) is a newly identified lncRNA with critical implications in cancer biology, first recognized in gastric cancer. It is located on chromosome 2q33.3, spanning 87,324 base pairs. It is further flanked by the protein-coding genes *KLF7* (Kruppel-like factor 7) and *CREB1* (CAMP Responsive Element Binding Protein 1), which exhibit overlapping expression profiles. Additionally, its locus overlaps with the oppositely transcribed lncRNA *LOC101927865* and shares an exonic sequence. *MYOSLID* is classified as a natural antisense lncRNA and plays a regulatory role in the Serum Response Factor (SRF)-dependent pathway, interacting with myocardin (MYCOD). *MYOSLID* is recognized as the first human vascular smooth muscle cell (VSMC)-selective modulator, enhancing VSMC differentiation via cooperative actions with MKL1 (MYCOD-Related Transcription Factor A). It also modulates the transforming growth factor (TGF-β) signaling pathway, specifically through TGF-β/SMAD signaling [12]. While *MYOSLID*’s functional role in various cancers, including colorectal cancer (CRC) [13], gastric cancer (GC) [6], osteosarcoma (OS) [8], head and neck squamous cell carcinoma (HNSCC) [7], and oral cell squamous carcinoma (OSCC) [9], is evident, as illustrated in Figure 1, further exploration of *MYOSLID’s* role in other cancers is necessary.

Therefore, in this study, we have discussed the mechanistic and regulatory roles of *MYOSLID* and uncovered the intricate connections in various cancers, as it is poised to serve as a promising emerging biomarker. Our focus is revealing *MYOSLID*’s multifaceted roles in cancer development, progression, and diagnosis. *MYOSLID* drives cancer progression by disrupting critical signaling pathways that govern cell survival, proliferation, and metastasis. Furthermore, this study delves into the mechanistic and regulatory functions of *MYOSLID* in modulating key cancer hallmarks and cellular phenotypes, including invasion, epithelial–mesenchymal transition (EMT), metastasis, and proliferation, particularly through the sequestration of miR-29c-3p (microRNA-29c-3 prime). We have also highlighted the involvement of various signaling pathways, such as Mincle signaling, apoptotic signaling, and the PD-L1 (Programmed Death-Ligand 1) pathway. Pathway analysis, including Gene Ontology and various bioinformatics tools, was employed to identify relevant pathways alongside Kaplan–Meier survival and differential expression analyses.

## 2. Literature Search Methodology

This literature has explored recent insights into *MYOSLID*, a long non-coding RNA (lncRNA) implicated in cancer biology. An extensive literature search was conducted across reputable databases, including ScienceDirect, PubMed, Web of Science, and DOAJ (Directory of Open Access Journals), until January 2025, highlighting a striking gap in research on *MYOSLID*. We strategically combined the specific keyword *MYOSLID* with terms such as cancer, proliferation, metastasis, invasion, immune response, and cellular energetics. Two independent researchers (K.M and S.M) systematically performed the literature search, followed by regular consultations with an experienced researcher (A.J) specialized in cancer biology. This collaborative approach strengthened our findings by providing a rich context on *MYOSLID* and its various roles in cancer. Despite extensive searches across prominent databases, only nine relevant papers were identified, highlighting the scarcity of research on *MYOSLID*. This significant gap serves as the rationale behind our initiative to write this article, aiming to bridge the void and contribute to this unexplored area. Our work addresses this unmet need and provides a foundation for future research on *MYOSLID*. An in-depth analysis was performed, focusing on key aspects such as lncRNAs that were being found, dysregulated genes or proteins, experimental techniques that have been employed, cell lines that have been used, and clinicopathological features that were assessed.

This review aligns with the framework of cancer hallmarks articulated by Douglas Hanahan and Robert Weinberg in their influential paper published on 1 January 2022. Of the identified hallmarks, we focused our study of *MYOSLID* on four aspects: sustaining proliferative signaling, avoiding immune destruction and cell death resistance, activating invasion and metastasis, and deregulating cellular metabolism [14].

## 3. Role of *MYOSLID* in Different Cancer Hallmarks

**Figure 2:**
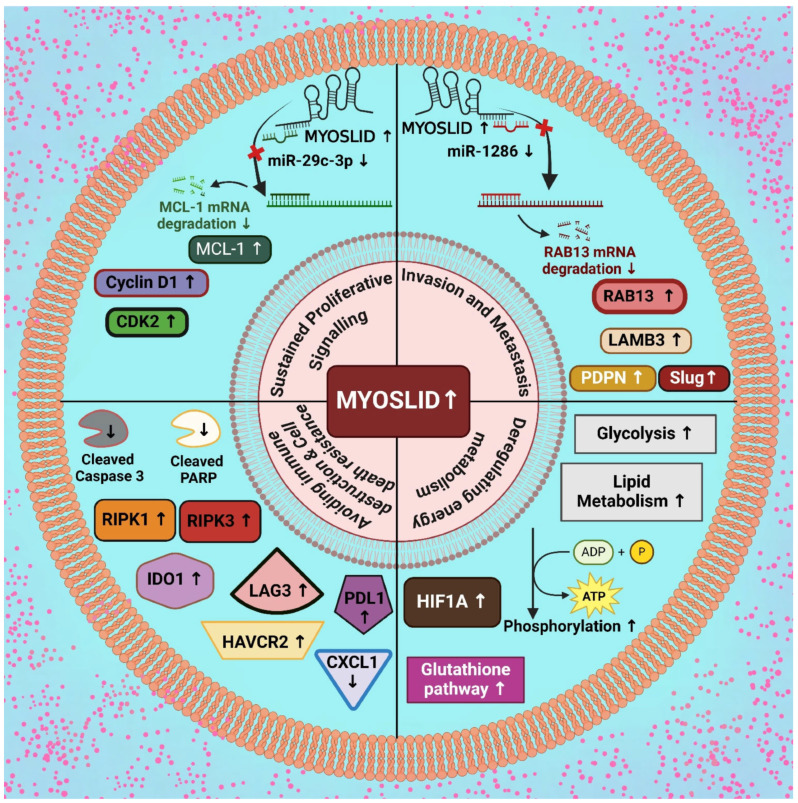
Upregulatory effect of *MYOSLID* in modulation of various cancer hallmarks. [Note: ↑—upregulation, ↓—downregulation] (Created with BioRender.com).

The expression patterns of *MYOSLID* are often altered across various types of cancer. They play a role in processes like sustained proliferative signaling, invasion facilitation, metastasis, immune cell dysregulation, cell death resistance, and cellular energetics. In the following section, we will discuss each of these processes individually (Figure 2).

### 3.1. MYOSLID and Sustaining Proliferative Signaling

Cell proliferation is a fundamental process in cancer progression, characterized by uncontrolled cell division and growth that drives tumor expansion. The capacity of cells to maintain proliferative signaling is mediated through diverse mechanisms [15]. Researchers have identified numerous lncRNAs upregulated in tumor samples (CRC patients), including *LINC02257*, *LINC02188*, *MYG1-AS1*, *C6orf223*, *L-SKA21*, and *MYOSLID*. Among these, *MYOSLID* remains relatively unexplored, prompting our investigation into its potential role in CRC. Expression levels were examined across multiple CRC cell lines—HCT116, HCT15, HCT8, DLD1, SW480, RKO, LOVO, CaCO2—and a normal colonic epithelial cell line (NCM460). HCT15 cells exhibited the highest expression of *MYOSLID*, with a fold change of approximately 13.00 (*p* < 0.05) relative to other colon cancer cell lines.

*MYOSLID* expression was detected under mechanistic conditions, such as hypoxia, defined by reduced oxygen availability. Two small interfering RNAs (siRNAs) were transfected into HCT15 cells to diminish the expression of *MYOSLID*, resulting in a significant decrease in the proliferative capacity. Additionally, transwell and wound healing assays indicated that inhibition of *MYOSLID* in HCT15 colorectal cancer cells by applying siRNAs led to a substantially decreased ability to proliferate. *MYOSLID* is a well-established regulator of cancer progression, primarily through its role in stabilizing hypoxia-inducible factor 1-α (HIF1A) [2].

Under normoxic conditions, HIF-1α is tightly regulated by hydroxylation mediated by prolyl hydroxylase domain (PHD) enzymes, which utilize oxygen as a substrate. This hydroxylation targets HIF-1α for recognition by the von Hippel–Lindau (VHL) ubiquitin ligase complex, resulting in proteasomal degradation. In contrast, under hypoxic conditions, the limited availability of oxygen reduces PHD activity, thereby preventing the hydroxylation and degradation of HIF-1α. Thereafter, this stabilized form of HIF-1α accumulates within the nucleus. Notably, HIF-1α interacts with minichromosome maintenance (MCM) proteins, such as MCM2, MCM3, MCM5, and MCM7, which are crucial for DNA replication licensing. This interaction inhibits MCM-driven DNA replication, thereby linking HIF-1α activity to cell cycle control and contributing to the regulation of cell proliferation under hypoxia [16,17]. The regulation of HIF-1α is further influenced by cyclin-dependent kinases (CDKs) and chaperone-mediated autophagy (CMA). CDK1 activity promotes HIF-1α stabilization, whereas CDK2 activity promotes its degradation through CMA during the s-phase of the cell cycle. The inhibition of CMA prevents and facilitates the stabilization of HIF-1α, leading to its accumulation, which subsequently induces cell cycle arrest and halts cellular proliferation. Additionally, MCM proteins are present in excess compared to the number of replication origins, with significant reductions in MCM protein levels up to 90% having minimal impact on DNA replication under normal conditions. However, during hypoxia, reduced levels of MCM7 result in the increased stabilization of HIF-1α, which slows DNA replication but allows for the selective replication of essential genomic regions required for cell survival under stress [17]. However, HIF-2α, a paralog of HIF-1α, has been shown to synergize with c-Myc, a critical oncogene, to enhance cell proliferation and tumor progression. HIF-2α enhances Myc function by activating specific glycolytic genes responsible for cancer growth [18].

Among four analyzed CRC cell lines (LOVO, HCT116, RKO, SW480), RKO cells exhibited the highest expression level of *MYOSLID*, where these cells were selected to generate a stable *MYOSLID* knockdown cell line. One specific knockdown construct, *shMYOSLID* (Short Hairpin RNA targeting *MYOSLID*), suppressed *MYOSLID* expression significantly and was therefore utilized for further experiments.

Knockdown of MYOSLID significantly inhibited the proliferation of RKO cells, as evidenced by the results of the CCK-8 assay. In addition, the EdU (5-Ethynyl-2′-deoxyuridine) incorporation assay revealed a marked reduction in cell growth following *MYOSLID* depletion. Finally, the colony formation assay demonstrated a diminished ability of *MYOSLID* knockdown cells to form colonies [13]. Collectively, these findings suggest that depletion of *MYOSLID* inhibits CRC cell proliferation. Subsequent investigations have identified *MYOSLID* as a core lncRNA within a regulatory model related to necroptosis. To elucidate its role, inhibitors targeting distinct cell death pathways were employed, including apoptosis, autophagy, necroptosis, and ferroptosis. It was observed that both the apoptosis inhibitor Q-VD-OPH (quinolyl-valyl-O-methylaspartyl-[-2,6-difluorophenoxy]-methyl ketone) and the necroptosis inhibitor Necrostatin-1 partially rescued the proliferative ability of *MYOSLID* knockdown cells. Further, verification using the calcein-AM/PI assay demonstrated that necrostatin-1 partially restored the proliferation of cells with *MYOSLID* depletion. These results indicate that *MYOSLID* facilitates CRC cell growth by suppressing the necroptosis pathway [13].

Necrostatins, a class of small-molecule inhibitors targeting receptor-interacting protein kinase 1 (RIPK1), play a crucial role in modulating necroptosis, a programmed necrotic cell death. By selectively inhibiting the kinase activity of RIPK1, it prevents the formation of the necrosome complex, disrupting the downstream activation of MLKL (Mixed Lineage Kinase Domain-Like Pseudokinase) and RIPK3 (Receptor Interacting Serine/Threonine Kinase 1). This prevents the rupture of plasma membranes and the subsequent release of damage-associated molecular patterns (DAMPs) by inhibiting the execution of necroptosis, thereby activating inflammatory pathways. Furthermore, it contributes to immune modulation by restricting the recruitment of pro-tumorigenic immune cells, including myeloid-derived suppressor cells (MDSCs) and tumor-associated macrophages (TAMs). MDSCs and TAMs are critical mediators of tumor progression, angiogenesis, and metastasis [19].

Moreover, necrostatins inhibit the activation of nuclear factor kappa-light-chain-enhancer of activated B cells (NF-κB), a pivotal downstream pathway of RIPK1. NF-κB is a key regulator of genes involved in cell survival, proliferation, and immune evasion. By inhibiting NF-κB signaling, necrostatins reduce the transcription of pro-survival and anti-apoptotic genes, alleviating apoptotic pressure on tumor cells. This suppression of apoptotic pathways promotes prolonged tumor cell proliferation by enabling tumor cells to evade intrinsic and extrinsic death signals [19].

Necrostatins disrupt the immunogenic properties of necroptosis by inhibiting the release of DAMPs and pro-inflammatory cytokines, which are essential for activating dendritic cells (DCs) and subsequent antigen cross-presentation. The suppression of immunogenic cell death (ICD) impairs the activation of cytotoxic CD8+ T lymphocytes, weakening the adaptive immune response against tumor cells. This immunosuppressive effect further facilitates immune evasion, allowing tumor cells to persist and proliferate in a hostile immune microenvironment [19].

In recent studies, Zhong et al. (2022) [2] performed GSEA to investigate pathway activities between high-risk and low-risk groups. The five most significantly enriched pathways in each group were identified based on the criteria of *p* < 0.05, q < 0.25, and |NES| > 1.5. The high-risk group showed enrichment in pathways such as hallmark hypoxia through gene set enrichment analysis. Simultaneously, hallmark pathways associated with oxidative phosphorylation and Myc targets were found to be enriched in the low-risk group.

Applying immune-related algorithms showed distinct infiltration patterns of immune and stromal cells in 500 CRC patients. The ESTIMATE algorithm revealed higher stromal, immune, and ESTIMATE scores in the high-risk group, suggesting a more tumor-supportive microenvironment. *MYOSLID*, known to promote EMT and tumor invasion, contributes to changes by influencing stromal activation and immune cell recruitment. CIBERSORT analysis revealed proportions of M0 macrophages, activated mast cells, and regulatory T cells in the high-risk group.

Conversely, populations of activated and resting dendritic cells, plasma cells, and CD4 memory T cells were reduced. Risk scores positively correlated with M0 macrophages and negatively with resting CD4 memory T cells, consistent with a tumor-promoting immune landscape influenced by hypoxia and *MYOSLID*-driven pathways. The analysis of cancer-associated fibroblasts (CAFs) across multiple algorithms, including XCELL, TIMER, QUANTISEQ, MPCOUNTER, and EPIC, showed a strong positive correlation between risk scores and CAFs, suggesting *MYOSLID* promotes stromal remodeling to support tumor growth [2]. Mechanistically, *MYOSLID* appears to sustain cellular growth by interacting with RNA-binding proteins and microRNAs, thereby influencing the post-transcriptional regulation of genes associated with proliferation. For example, *MYOSLID* functions as a competing endogenous RNA (ceRNA) by acting as a molecular sponge for miR-29c-3p in gastric cancer. In typical cellular processes, miR-29c-3p interacts with complementary sequences within the 3′-untranslated region (3′-UTR) of target mRNAs, including MCL-1, leading to mRNA degradation or translational inhibition. However, *MYOSLID* sequesters miR-29c-3p by harboring binding sites complementary to the miRNA, reducing its availability to target MCL-1 (Figure 2).

Further, MCL-1 encodes an anti-apoptotic protein that promotes cell survival and inhibits programmed cell death (apoptosis). Its overexpression, facilitated by *MYOSLID*’s sponging effect, enhances gastric cancer growth. By binding miR-29c-3p and preventing its interaction with MCL-1 at the 3′-UTR, *MYOSLID* disrupts the normal miRNA-mediated regulation of this oncogene, promoting tumor progression [6]. Han et al. (2019) conducted a comprehensive study on the role of miR-29c-3p in modulating the oncogenic effects of lncRNA *MYOSLID* in gastric cancer (GC) cells. Their study transfected miR-29c-3p mimics into the GC cell line BGC-823, engineered to overexpress lncRNA *MYOSLID*. The results showed that the proliferation of GC cells was significantly increased due to the overexpression of lncRNA *MYOSLID*. However, this proliferation was partially inhibited upon transfection with miR-29c-3p mimics.

Furthermore, in vivo experiments involving mice injected with these cells demonstrated tumor formation in all groups. Notably, the size and weight of the tumors in the group overexpressing miR-29c-3p mimics were significantly reduced compared to the empty vector control group [6]. Moreover, overexpression of miR-29c-3p resulted in decreased Ki-67-positive cells in a xenograft mouse model, indicating reduced cellular proliferation and an increased proportion of apoptotic cells. The reduced level of MCL-1′s role in GC cell lines SGC-7901 and BGC-823 was studied through a comprehensive investigation by Western blot and qRT-PCR analysis, where siRNA was transfected into these cells to study knockdown MCL-1 expression effectively. Functional assays and flow cytometry revealed that decreased MCL-1 expression inhibited cancer cell growth and colony formation [6]. Further, the knockdown of MCL-1 effectively induced cell cycle arrest, accompanied by a significant downregulation of cell cycle-related proteins, including CDK2 (Cyclin-Dependent Kinase 2) and Cyclin D1 (Figure 2). Simultaneously, apoptosis-related proteins, such as cleaved caspase-3 and cleaved PARP, exhibited substantial upregulation [6]. Gong et al. (2024) conducted experimental validations on three cancer-related lncRNAs (CRLs): *FAM27E3* (Family with Sequence Similarity 27 Member E3), *LINC02367*, and *MYOSLID* on OSCC cell lines SCC25 and HN30. Using qRT-PCR analysis, they confirmed a significant reduction in the expression levels of these CRLs, including *MYOSLID*, following siRNA-mediated knockdown compared to control cells [9]. Functional assays revealed that *MYOSLID* is critical in regulating OSCC cell proliferation. Specifically, CCK-8 assays demonstrated a marked reduction in cell proliferation and viability in *MYOSLID* knockdown cells compared to controls. Furthermore, Edu staining showed a significant decrease in the proportion of Edu-positive cells in the *MYOSLID* knockdown group for both HN30 and SCC25 cells, indicating reduced DNA synthesis and cell replication. Colony formation assays further confirmed that the knockdown of *MYOSLID* significantly inhibited the long-term proliferative capacity of OSCC cells. These findings collectively highlight the pivotal role of *MYOSLID* in promoting OSCC cell proliferation [9].

Researchers synthesized antisense oligonucleotides (ASOs) to target and suppress *MYOSLID* expression in CRC cell lines HCT116 and RKO. The suppression of *MYOSLID* significantly inhibited cancer cell activity and proliferation, corroborating findings from previous studies. Additionally, the expression levels of key markers associated with proliferation and senescence were analyzed. HCT116 cells showed a marked upregulation of Ki67 and MCM2 following *MYOSLID* knockdown. Similarly, RKO cells showed increased Ki67, Lamin B1, and MCM2 levels, while the expression of p16, a senescence-associated marker involved in cell cycle regulation, was notably reduced [20]. Cumulative studies, taken together, suggest that *MYOSLID* is the key regulator for sustained proliferative signaling in various cancer cells targeting miRNAs, hypoxia-related factors, and immune cells.

### 3.2. MYOSLID in Avoiding Immune Destruction and Cell Death Resistance

Immune function dysregulation and resistance to cell death are cancer hallmarks that allow tumor progression by escaping programmed cell death pathways [14]. These mechanisms are critical for maintaining homeostasis and protecting the human body by eliminating abnormal cells, including cancer cells. However, numerous malignancies have evolved mechanisms to evade these cell death programs. Such evasion is mediated by the dysregulation of key factors, including unchecked tumor cell proliferation, metastasis, and resistance to apoptosis. Emerging studies suggest various forms of programmed cell death, including autophagy, necrosis, and apoptosis, are intricately associated with cancer cell sensitivity to immunotherapy [19]. Moreover, resistance to these pathways unveils a substantial challenge in the effective treatment of cancer. The interplay of apoptosis resistance in cell cycle regulation significantly contributes to tumor cell survival [21].

Recent investigations have focused on the role of *MYOSLID* in modulating cell death resistance. Using flow cytometry and Western blot analysis, researchers examined the role of *MYOSLID* in gastric cell lines SGC-7901 and BGC-823 and observed significant effects following its knockdown. The study further revealed that the depletion of *MYOSLID* led to increased expression of apoptosis markers, such as cleaved PARP and cleaved caspase-3 (Figure 2). The critical cell cycle regulators, such as cyclin D1, CDK2, cyclin D3, and CDK4, were reduced in cells upon *MYOSLID* knockdown. Research has shown that regulators of necroptosis, such as RIPK3, RIPK1 (Receptor Interacting Serine/Threonine Kinase 1), and MLKL, play an essential role in modulating immune tumor response. These molecules are especially involved in activating CD8+ and CD4+ activation pathways to enhance the immune response by increasing CD4+ and CD8+ T cells [6].

In in vitro studies on transgenic mouse models, RIPK1 expression is upregulated in cells influenced by *MYOSLID*, leading to chronic inflammation. This chronic inflammatory state is associated with increased tumorigenesis [22]. Activated RIPK1 activates RIPK3, leading to the autophosphorylation of RIPK3 (Figure 2) [23]. Research has indicated that TRADD (Tumor Necrosis Factor Receptor type1-associated Death Domain Protein), a death domain adaptor protein, can directly interact with RIPK3, even without RIPK1 in specific cell types. This interaction enhances the induction of necroptosis via an alternative pathway activated by tumor necrosis factor (TNF). Moreover, TRADD can activate FADD (Fas-associated death domain), which subsequently leads to the activation of procaspase 8. This activation results in the formation of procaspase heterodimers, which are ultimately degraded by the action of RIPK1 and RIPK3. This mechanism contributes to the resistance of cells to apoptotic signals, thereby influencing cell survival [24].

Moreover, CXCL1 (C-X-C motif chemokine ligand 1) and Mincle signaling pathways also contribute to the activation of immune cells, as seen upon the knockdown of *MYOSLID*. The elevation of CXCL1 was found to promote the migration of M2 TAMs while simultaneously disrupting the aggregation of CD4+ and CD8+ T cells within tumor microenvironments (Figure 2). Mechanistic investigations revealed that CXCL1 activates the NF-κB signaling pathway [25].

Researchers in previous studies investigated the expression of immune checkpoints, which are regulatory proteins of the immune system, and their association with specific lncRNAs divided into two CRC subgroups named cluster 1 and cluster 2. Patients in cluster 2 exhibited significantly higher levels of key immune checkpoint proteins, including LAG3 (Lymphocyte-Activation Gene 3) (*p* < 0.05), IDO1 (Indoleamine 2,3-dioxygenase 1), HAVCR2 (Hepatitis A virus cellular receptor 2), and PD-L1, compared to cluster 1 (Figure 2). This elevated expression suggested cluster 2 patients may respond favorably to immune checkpoint inhibitors. Among the studied lncRNAs, *SNHG26* (Small nucleolar RNA host gene 26), *NSMCE1-DT* (NSMCE1 Divergent Transcript), *LINC02084*, *PCED1BAS1*, *MYOSLID*, *LINC02428*, *LINC01857*, *LINC00861*, *ATP2B1-AS1* (ATPase plasma membrane Ca2+ transporting 1 antisense RNA 1), *LINC02381*, *PRKAR1B-AS2* (Protein Kinase CAMP-Dependent Type I Regulatory Subunit β long noncoding RNA), *MRPS9-AS1* (MRPS9 Antisense RNA 1), and *LINC01679* showed a positive correlation with immune checkpoints, particularly PD-L1, indicating that their increased expression enhances PD-L1 levels. This activity suppresses T cell-mediated immune responses against tumors by inhibiting their apoptotic pathways, allowing cancer cells to evade destruction by the immune system.

By promoting the expression of PD-L1, *MYOSLID* indirectly supports tumor immune evasion mechanisms; conversely, other lncRNAs, such as *STAG3L5P-PVRIG2P-PILRB* (Stromal Antigen 3-Like 5 Pseudogene-Poliovirus Receptor-Related Immunoglobulin Domain-Containing 2 Pseudogene-Paired Immunoglobulin-Like Type 2 Receptor β) and *LENG8-AS1* (Leukocyte Receptor Cluster Member 8 Antisense RNA 1), exhibited a negative correlation, implying reduced activity as PD-1 levels increased. While both clusters showed a similar composition of immune cells, Cluster 2 displayed a more significant infiltration of these cells into the tumor microenvironment, reflecting a more active immune state. Immune checkpoint inhibitors that block PD-1 or PD-L1 pathways can counteract these evasion mechanisms, reactivating apoptotic pathways in cancer cells [21]. The interaction between Programmed Death 1 (PD-1) and PD-L1 induces the activation of Src Homology Region 2 Domain-Containing Phosphatases (SHP2s) by tumor-infiltrating T cells (TILs). This activation suppresses T cell receptor signaling, adversely affecting the overall function of the immune system [26,27]. Han et al. (2024) demonstrated that in vitro knockdown of *MYOSLID* induced cell cycle arrest and apoptosis in gastrointestinal cancer cells at the G1 phase [28].

To further investigate the impact on the immune microenvironment and genetic alterations in colon cancer, researchers employed the maftools R package to analyze mutation profiles in high-risk and low-risk groups as stratified by the CSRL (Cellular Senescence-Related Long Non-Coding RNA) prognostic model. The high-risk group exhibited an elevated median mutation frequency relative to the low-risk group (116 vs. 102.5). Notably, the missense mutations were found to be the most prevalent type, dominating the classification of the single nucleotide variant (SNV) class. Further analysis of the mutated genes indicated that the APC gene had a higher mutation frequency in the low-risk group (79%) than the high-risk group (48%). The increased mutational burden observed in the high-risk group suggested a dysregulated apoptotic response, possibly driven by altered senescence regulation. Specifically, *MYOSLID*, an important lncRNA identified in the CSRL model, promoted cell survival and proliferation by suppressing apoptotic signaling pathways [20].

### 3.3. MYOSLID in Activating Invasion and Metastasis

Metastasis, which refers to the dissemination of cancer cells from primary tumors to regional lymph nodes or distant organs, plays a key factor in driving cancer progression and impacting patient prognosis. This intricate, multistep process is driven by cellular mechanisms such as EMT and partial epithelial–mesenchymal transition (p-EMT), which facilitate tumor cell migration, invasion, and colonization. Among the various hallmarks of cancer, invasion and metastasis are critical processes influenced by *MYOSLID*. The expression level of *MYOSLID* is markedly elevated in the Cal27 HNSCC cell line, as it shows co-expression of p-EMT markers such as (Podoplanin) PDPN and (Laminin Subunit β 3) LAMB3. Moreover, classical EMT markers, like E-cadherin and Vimentin, suggest that the Cal27 cell line closely resembles p-EMT [7].

To check the *MYOSLID* knockdown expression in these cell lines, two siRNA sequences, *MYOSLID-homo-96* and *MYOSLID-homo-232*, were selected based on their higher knockdown efficiency than *MYOSLID-homo-696*. Functional assays were conducted, which demonstrated that knockdown of *MYOSLID* significantly inhibited cellular migration and invasion. Wound healing assays revealed a significant reduction in the 48 h healing rate (*p* < 0.05), and a transwell assay showed that the cells migrating the Matrigel-coated filters in the siRNA-treated groups were significantly reduced compared to negative control (*p* < 0.05) [7]. Researchers further investigated the role of *MYOSLID* in OSCC by assessing the expression levels of p-EMT (Partial Epithelial-to-Mesenchymal Transition) markers PDPN, Slug (Snail Family Transcriptional Repressor 2), and LAMB3 and classical EMT markers Vimentin and E-cadherin following *MYOSLID* knockdown in OSCC cell lines. Results revealed that silencing *MYOSLID* substantially decreased the mRNA and protein concentration levels of Slug, PDPN, and LAMB3 compared to the control group, indicating its role in promoting the p-EMT process. However, the expression profiles of classical EMT markers, Vimentin and E-cadherin (Epithelial Cadherin), remain unaffected [7].

PDPN, a transmembrane glycoprotein, functions at the molecular level by interacting with C-type lectin-like receptors (CLEC-2) on platelets, facilitating platelet aggregation. This interaction contributes to tumor embolism, immune evasion, and hematogenous metastasis by promoting the extravasation of cancer cells. Additionally, PDPN associates with ezrin, radixin, and moesin (ERM) proteins via its intracellular domain, activating Rho GTPases, which mediate cytoskeleton reorganization and enhance cellular motility. Furthermore, PDPN-induced platelet aggregation stimulates the release of growth factors, such as TGF-β, thereby promoting tumor cell invasion and metastatic progression. The phosphorylation of PDPN intracellular residues by protein kinase A (PKA) and cyclin-dependent kinase 5 (CDK5) regulates its role in cell motility. While non-phosphorylated PDPN promotes cell migration, the phosphorylation of these residues inhibits this process, suggesting a therapeutic target for modulating PDPN activity. Preclinical studies have demonstrated the efficacy of targeting PDPN to inhibit tumor progression and metastasis.

Strategies such as monoclonal antibodies, chimeric antigen receptor (CAR)-T cells, and synthetic molecules have shown significant inhibition of PDPN-mediated processes. For example, monoclonal antibodies like NZ-1 effectively disrupt PDPN-CLEC-2 (Podoplanin-C-Type Lectin-Like Receptor 2) interactions, reducing platelet aggregation and metastatic dissemination [29]. LAMB3 (Laminin Subunit β 3), as a component of laminin-332 (LM-332), plays a pivotal role in facilitating basement membrane (BM) degradation, a critical step in enabling tumor cells to invade surrounding tissues. By contributing to BM breakdown, LAMB3 allows tumor cells to breach this structural barrier, a key event in metastatic progression. LAMB3 interacts with extracellular matrix (ECM) components and cell surface receptors, such as integrins (e.g., ITGB1), to promote cell adhesion and migration. This interaction leads to cytoskeletal reorganization, strengthens adhesion within the tumor microenvironment, and facilitates the migration of highly metastatic cancer cells.

Additionally, LAMB3 activates intracellular signaling cascades, including the PI3K and MAPK (mitogen-activated protein kinase) pathways, which regulate critical processes such as cell survival, proliferation, and motility. These pathways are central to tumorigenesis and metastatic progression, enhancing cancer cells’ proliferative and invasive capacities. Overexpression of LAMB3 has been strongly associated with lymph node metastasis, where it enhances tumor cell dissemination by increasing motility and invasion, ultimately facilitating the establishment of secondary metastatic sites. Furthermore, LAMB3 interacts with proteins such as collagen VI and osteopontin, binding to integrins like ITGB1 (integrin subunit β 1), which supports ECM remodeling and promotes the migration and invasion of cancer cells [30].

RAB13 (Ras-related protein) is a well-known oncogene in OS, enhancing metastasis in epithelial cancers. *MYOSLID*-mediated overexpression of RAB13 further drives migration and invasion in osteosarcoma cells, highlighting its oncogenic role.

*MYOSLID* sequesters miR-1286, which suppresses metastasis-associated genes. This sponging activity prevents miR-1286 from downregulating its target, the oncogene RAB13, which is critical for cytoskeletal remodeling and cell motility. RAB13 is a well-established promoter of cellular migration and invasion. *MYOSLID* upregulates RAB13 by inhibiting miR-1286 activity, enhancing the metastatic potential of OS cells. When *MYOSLID* is silenced, miR-1286 levels increase, leading to reduced RAB13 expression and suppressed metastatic behavior. Studies further showed that the knockdown of *MYOSLID* significantly reduced OS cell migration and invasion. These effects were attributed to the *MYOSLID*/miR-1286/RAB13 (Myosin Light Chain Kinase-Interacting Protein/MicroRNA-1286/RAB13, Member RAS Oncogene Family) axis, as overexpressing RAB13 rescued the impaired migratory and invasive capacities of *MYOSLID*-deficient cells (Figure 2) [8]. Xiong et al. (2024) and other researchers investigated the expression of LAMB3, Slug, and PDPN genes in cancer cells, demonstrating that these genes are upregulated in HNSCC (Figure 2) [28]. Notably, silencing of *MYOSLID* resulted in downregulating these metastasis-associated genes. These findings highlight the pivotal role of *MYOSLID* in promoting metastasis by regulating the expression of key mediators involved in crucial progression, dissemination, EMT, and extracellular matrix remodeling [28].

### 3.4. MYOSLID and Deregulating Cellular Metabolism

Energy metabolism reprogramming is a hallmark of tumorigenesis, where imbalances in oncogenic signaling pathways disrupt the synthesis and metabolism of key biomolecules, including fatty acids, glucose, and glutamine, thus driving tumor cell proliferation. In this context, lipid metabolism-related lncRNA *MYOSLID* plays a critical role in cellular energetics. The clinical data of 446 colon cancer patients from TCGA (The Cancer Genome Atlas) were summarized. Through Spearman correlation analysis, 27 genes and 349 lncRNAs associated with lipid metabolism were identified [21]. Subsequently, eighteen lipid metabolism-related lncRNAs were significantly (*p* < 0.05) related to patient prognosis. These lncRNAs, *LINC02428*, *STAG3L5P-PVRIG2P-PVRIG2P-PILRB*, *NSMCE1-DT*, *MYOSLID*, *MRPS9-AS1*, *NCBP2-AS1*, *WARS2-AS1*, and *LENG8-AS1*, were found to be upregulated in tumor tissues. These eight lncRNAs and *LINC02084* have been previously associated with patient prognosis in hepatocellular carcinoma (HCC). Although the relationship between lncRNA expression and lipid metabolism in colon cancer has been rarely studied, it is essential to explore lipid metabolism-related lncRNAs using large-scale datasets. Christensen et al. (2022) demonstrated that the lncRNA *SNHG16* (Small Nucleolar RNA Host Gene 16) plays a role in the transcription of lipid metabolism-related genes and can target multiple microRNA families [21,31].

Yurui et al. (2021) developed a ceRNA network associated with fatty acid metabolism, suggesting that lncRNA-related lipid metabolism influences the prognosis of colon cancer. The upregulation of *MYOSLID* in tumor tissues indicates its potential role in reprogramming lipid metabolism, thereby affecting energy homeostasis with colon cancer cells, contributing to tumor progression, and potentially offering new avenues for individualized treatment strategies. Gene set enrichment analysis (GSEA), utilizing the Kyoto Encyclopedia of Genes and Genomes (KEGG) pathways, was conducted to identify biological functions and signaling pathways associated with different clusters. A comparative analysis of Clusters 1 and 2 revealed significant enrichment of pathways in Cluster 2, including the glutathione metabolism (*p* = 0.002), proline and arginine metabolism (*p* < 0.001), galactose metabolism (*p* = 0.002), citric acid cycle (TCA) (*p* = 0.001), oxidative phosphorylation (*p* < 0.001), and proteasome (*p* = 0.010) (as shown in Table 1). Patients in Cluster 2 exhibited improved survival outcomes, suggesting that the pathways enriched in this cluster may play a role in suppressing the initiation and progression of colon cancer (Figure 2) [21,32].

Hypoxia, a prevalent condition in solid tumors, arises due to the high oxygen demands of cancer cells and the disorganized structure of their vasculature. Under these conditions, cancer cells undergo metabolic reprogramming, shifting to glycolysis for energy production despite the availability of oxygen, a phenomenon known as the Warburg effect [2,33]. This metabolic adaptation enables the synthesis of essential biomolecules for proliferation and leads to increased lactate production, disrupting extracellular pH homeostasis. HIF-1 mediates the cellular response to hypoxia by promoting processes such as EMT, enhanced cell migration, invasion, and metabolic reprogramming. Furthermore, hypoxia and lncRNA *MYOSLID* interact in a regulatory network that significantly contributes to tumor progression. Experimental evidence revealed that *MYOSLID* is markedly upregulated in colorectal cancer cell lines, particularly in HCT15. Thus, induced *MYOSLID* expression suggests involvement in cancer cell survival, proliferation, and adaptation within hypoxia tumor microenvironments (Figure 2) [2]. HIF-1α plays a central role in cellular adaptation to hypoxia by orchestrating a metabolic switch from oxidative phosphorylation to glycolysis, enabling efficient ATP production in low-oxygen conditions. It does this by upregulating the expression of key glycolytic enzymes, including phosphofructokinase (PFK), hexokinase (HK), and pyruvate kinase M2 (PKM2), which promote the glycolytic pathway [34].

Additionally, HIF-1α enhances the expression of lactate dehydrogenase A (LDHA), which facilitates the conversion of pyruvate to lactate, allowing glycolysis to continue even in the absence of adequate oxygen. To further support glycolysis, HIF-1α induces the expression of glucose transporters, such as GLUT1 and GLUT3, increasing glucose uptake to meet cellular energy demands. Simultaneously, HIF-1α downregulates oxidative phosphorylation by inducing pyruvate dehydrogenase kinase 1 (PDK1). This inhibits pyruvate dehydrogenase (PDH) and limits pyruvate entry into the TCA cycle. This shift reduces mitochondrial oxygen consumption and decreases the production of reactive oxygen species (ROS), mitigating potential oxidative damage under hypoxic conditions. Furthermore, HIF-1α promotes glycogen synthesis, creating an energy reservoir to sustain cells during prolonged periods of oxygen deprivation. The increased lactate production resulting from heightened glycolysis leads to acidification of the extracellular environment, thereby supporting tumor cell survival, proliferation, and invasion as well as contributing to tumor progression and adaptation to hypoxia [35].

## 4. Survival Analysis and Prognostic Value of *MYOSLID* in Cancers

We have obtained data from TCGA (https://portal.gdc.cancer.gov/) and the ENCORI (https://rnasysu.com/encori/) database accessed on 4 December 2024 to study the prognostic significance of *MYOSLID* expression across various cancer types. Cancer patients were grouped into high and low *MYOSLID* expression level cut-off values of *MYOSLID* expression levels. Kaplan–Meier survival analysis and log-rank tests were employed to evaluate these groups’ overall survival differences.

**Figure 3:**
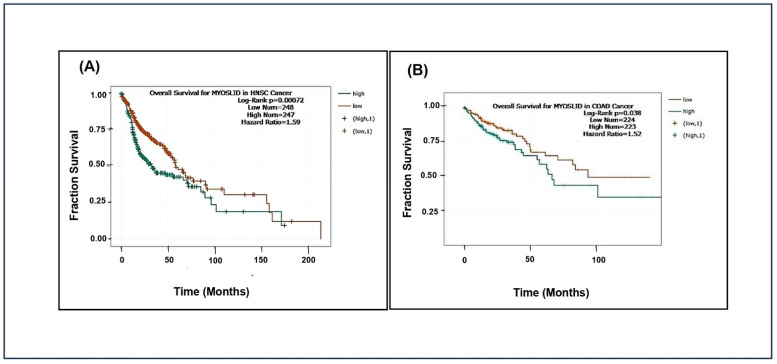
Data plots of Kaplan–Meier estimate overall survival of a group of patients retrieved from TCGA database (https://ualcan.path.uab.edu/, accessed on 4 December 2024): (**A**) head and neck squamous cell carcinoma; (**B**) colon adenocarcinoma (COAD).

The analysis showed that patients with high *MYOSLID* expression exhibited significantly lower median overall survival than those with low expression in head and neck squamous cell carcinoma (HNSCC) and colon adenocarcinoma (COAD). However, *MYOSLID* expression levels did not significantly stratify survival outcomes in sarcoma (SARC) and stomach adenocarcinoma (STAD). We made Kaplan–Meier plots for HNSCC (*p* = 0.00072), SARC (*p* = 0.47), STAD (*p* = 0.2), and COAD (*p* = 0.038). It was observed that hazard ratios of 1.59 (Figure 3A) and 1.52 (Figure 3B) in patients of HNSCC and COAD with high *MYOSLID* expression lead to less survival compared to SARC and STAD patients with low expression. For example, HNSCC patients survived approximately 160 months compared to SARC patients, who survived more than 200 months. These data suggest that *MYOSLID* expression levels significantly correlate with poor prognosis and can be used as an independent prognostic marker.

**Figure 4:**
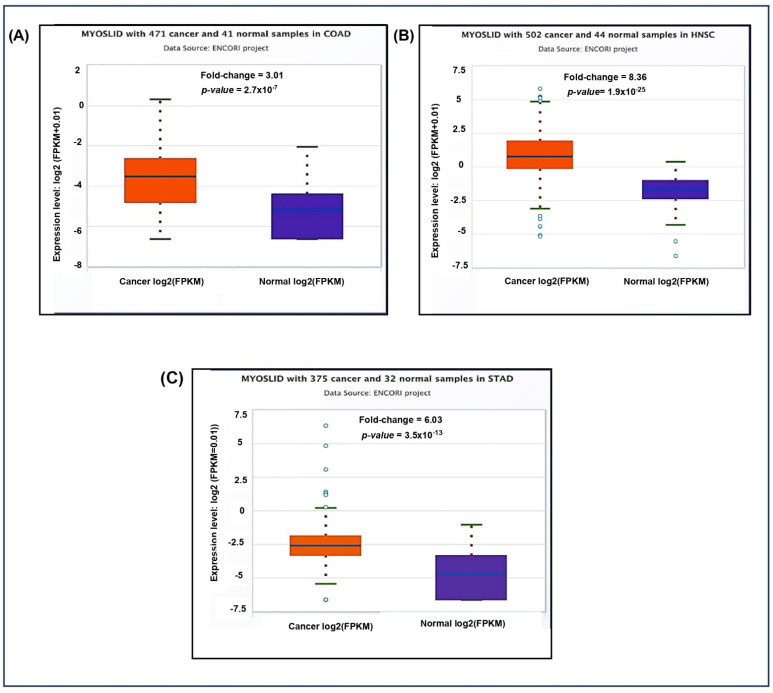
Differential expression of LncRNA *MYOSLID* in various cancer in the ENCORI database on 4 December 2024. (**A**) Colon adenocarcinoma (COAD). (**B**) Head and neck squamous cell carcinoma (HNSC). (**C**) Stomach Adenocarcinoma (STAD).

Additionally, the pan-cancer differential expression analysis showed significant upregulation of *MYOSLID* in multiple cancer types compared to normal tissues (Figure 4A). The fold change in COAD was 3.01, indicating higher expression in tumor tissues. Similarly, elevated expression levels were noted in (Figure 4B) HNSC 8.36 and (Figure 4C) STAD 6.03 compared to normal tissue counterparts.

## 5. *MYOSLID* Roles in Various Biological and Cellular Pathways

To study the potential molecular targets and pathways associated with *MYOSLID*, we have conducted a Gene Ontology (GO) pathway analysis. GO analysis elucidates the gene and its associated products, emphasizing key aspects such as biological processes, molecular functions, and KEGG pathways. The *MYOSLID* gene sequence was assessed using TargetScan (https://www.targetscan.org/vert_80/) (Ensemble 75) and subjected to functional annotation using the DAVID (https://davidbioinformatics.nih.gov/) (Database for Annotation, Visualization, and Integrated Discovery) bioinformatics tool. Results were visualized using GraphPad Prism (v10.1), where biological processes and pathways were plotted on the y-axis, and the negative logarithm of the false discovery rate, i.e., log10 FDR (false discovery rate), was represented on the x-axis. We focused on the top 10 enriched K terms in each category.

**Figure 5:**
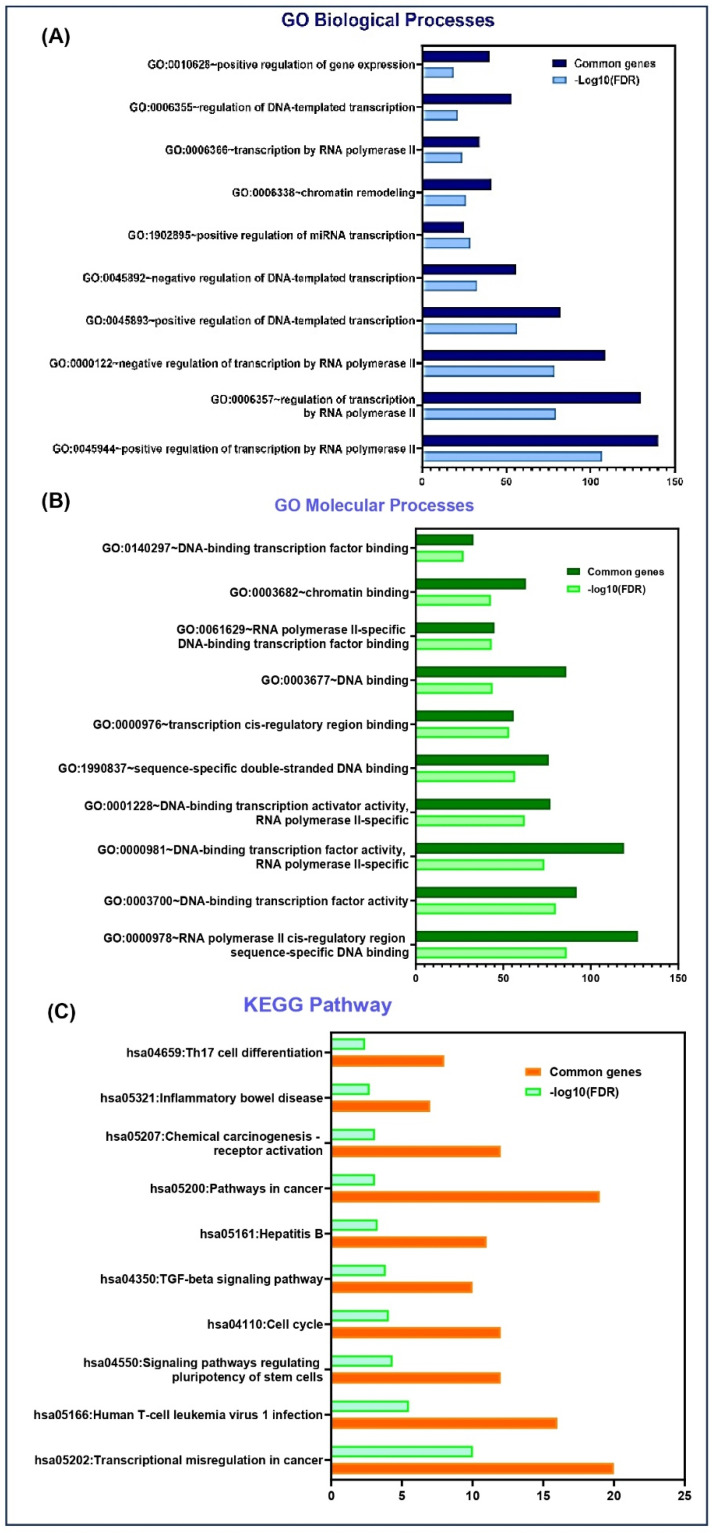
DAVID functional enrichment analysis of *MYOSLID* target genes in various biological processes and signaling pathways. (**A**) Significantly enriched GO biological terms. (**B**) Significantly enriched GO processes. (**C**) Significantly enriched KEGG pathway.

*MYOSLID* targets revealed their involvement in several biological processes, including (Figure 5A) the positive regulation of gene expression, the regulation of DNA-templated transcription, and transcription by RNA polymerase II. This reinforces the tumorigenic functions of *MYOSLID*, which were demonstrated through experiments of *MYOSLID* binding to various miRNAs such as miR-29c-3p and miR-1286 [6].

Furthermore, these targets also engage in molecular functions, (Figure 5B) including DNA binding (such as transcription cis-regulatory region binding, RNA polymerase II cis-regulatory region sequences) and sequence-specific double-stranded DNA binding.

In addition, KEGG pathway analysis highlighted the participation of *MYOSLID* target genes in cancer-related pathways (Figure 5C) such as chemical carcinogenesis through receptor activation, the TGF-β signaling pathway, and transcriptional misregulation in cancer. GO results may help in the future development of biomarkers linked with *MYOSLID*.

## 6. Conclusions and Future Outlook

Despite global advancements in developing novel diagnostic and prognostic approaches for cancer treatment, long non-coding RNAs (lncRNAs) have attracted significant attention in recent years due to their essential roles in cancer biology. Cell proliferation, metastasis, angiogenesis, and tumor growth suppression are cellular processes where these molecules function as key regulators. LncRNAs profoundly influence how they function in various biological activities by regulating gene expression at epigenetic, transcriptional, and post-transcriptional levels. Among these, elevated levels of *MYOSLID* in various tumor tissues highlight its importance in the progression and pathogenesis of cancer.

Evidence suggests that *MYOSLID* plays a pivotal role in driving tumor progression by modulating EMT, a process that enhances the metastatic potential of cancer cells by enabling them to adopt mesenchymal traits, thereby promoting their migratory and invasive abilities as tabulated in Table 1. Furthermore, *MYOSLID* is intricately linked to several critical signaling pathways, most notably the PI3K/AKT pathway, which is essential for regulating cell survival, growth, metabolism, tumor progression, and actin cytoskeleton remodeling, which further highlights its significance in maintaining cellular architecture and enabling motility, both of which are vital for tumor invasion and metastasis.

*MYOSLID* is implicated in necroptosis, a programmed cell death, which is associated with tumor progression. The downregulation of *MYOSLID* correlates with increased immune cell infiltration, especially the elevation of CD4+ and CD8+ T cell populations, indicating its potential role in modulating the tumor immune microenvironment. RAB13 is a key promoter of cellular migration and invasion, and *MYOSLID* upregulates RAB13 by inhibiting miR-1286 activity, thereby enhancing the metastatic potential of osteosarcoma (OS) cells. The Kaplan–Meier survival analysis curve, which we have obtained, shows that *MYOSLID* expression levels were comparatively lower in HNSC (*p* = 0.00072) than in other carcinomas analyzed. Further research suggests that *MYOSLID* promotes tumor cell proliferation and invasion, making it a hallmark of cancer and a defining factor in various cancer characteristics. Its multifaceted involvement in tumor biology makes *MYOSLID* a prospective diagnostic biomarker for early cancer detection and a potential target for therapeutic intervention. Targeting *MYOSLID* can potentially disrupt key mechanisms, such as EMT and immune evasion, thereby impairing tumor metastasis and progression. *MYOSLID* is a significant lncRNA in the realm of cancer biology, so understanding its role is imperative. Unfortunately, research on this critical factor is still scarce, with only a handful of experimental studies delving into its potential mechanical actions. This research gap additionally signifies the requirement for a detailed and comprehensive analysis of the regulatory mechanisms of *MYOSLID*.

Understanding the complex interactions of this pathway with key signaling networks and its implications in cancer progression is crucial for bridging existing knowledge gaps and facilitating transformative breakthroughs in cancer therapeutics. Despite the promising potential of lncRNAs in cancer therapeutics, several limitations hinder their clinical translation. A key challenge is their cellular penetration, as lncRNAs are highly susceptible to rapid degradation in the biological environment. Enhancing their stability and bioavailability requires the development of optimized chemical structures. Although lncRNAs can be readily synthesized and chemically modified to resist nucleotide degradation, their efficient delivery remains a significant obstacle. While liposomal delivery systems have been extensively explored for lncRNA transport, they face challenges related to scalability, reproducibility, chemical instability, and the risk of denaturation. Addressing these limitations is essential to fully unlock the therapeutic potential of lncRNAs and facilitate their safe and effective application in precision oncology [36]. Future research must prioritize unraveling its potential applications in precision medicine and targeted therapies, areas that hold promise for more personalized and effective treatment approaches. By deepening our analysis of *MYOSLID*’s contributions to cancer pathogenesis, its regulatory effects on tumor microenvironments, and its involvement in critical cellular functions like metastasis and immune modulation, we can unlock new possibilities for early detection and intervention. Ultimately, *MYOSLID* has the potential to emerge as a reliable therapeutic target, offering innovative strategies to disrupt cancer progression and paving the way for innovative diagnostic tools and therapeutic strategies. In the long term, *MYOSLID* holds immense promise as a reliable target in the fight against cancer, with the potential to significantly improve patient outcomes and advance cancer treatment paradigms.

## Figures and Tables

**Table 1:** Implications of *MYOSLID* with various hallmarks of cancer through targeting genes/proteins/pathways.

Hallmarks of Cancer	Cancer Types	Expression of *MYOSLID*	Clinicopathological Characteristics	*MYOSLID* Targets of Genes/Proteins/Pathways	References
Sustaining proliferative signaling	Colorectal cancer	↑	Gender, age, TNM stage, lymph node metastasis, distant metastasis, 5-year survival	HIF1A, CAFs, Necrostatin-1, Q-VD-OPH, *Ki67*, MCM2	[2,13,20]
	Gastric cancer	↑	-	miR-29c-3p (via sponging effect), MCL-1	[6]
	Osteosarcoma	↑	-	miR-1286/RAB13 signaling	[8]
	Oral squamous cell carcinoma	↑	-	Cuproptosis-related pathways involving *FAM27E3* and *LINC02367*	[9]
Avoiding immune destruction	Colorectal cancer	↑	Gender, age, TNM stage, lymph node metastasis, distant metastasis, 5-year survival	PD-L1, LAG3, IDO1, HAVCR2, CXCL1, and Mincle signaling pathways, RIPK1, RIPK3, MLKL	[21]
	Gastric cancer	↑	-	Cleaved caspase-3, cleaved PARP, cyclin D1, CDK2, CDK4, CDK6	[6]
Activating invasion and metastasis	Head and neck squamous cell carcinoma	↑	-	PDPN, LAMB3, Slug, Vimentin, E-cadherin	[7]
	Osteosarcoma	↑	-	RAB13, miR-1286	[8]
Deregulating cellular metabolism	Colorectal cancer	↑	Gender, age, TNM stage, lymph node metastasis, distant metastasis, 5-year survival	HIF1A, glycolysis-related metabolic pathways, Galactose metabolism, glutathione metabolism, proline/arginine metabolism, TCA cycle, oxidative phosphorylation	[21]

↑ Upregulation; HIF1A—Hypoxia-inducible factor α; CAFs—Cancer-Associated fibroblasts; Q-VD-OPH—quinolyl-valyl-O-methylaspartyl-[-2,6-difluorophenoxy]-methyl ketone; MCM2—Minichromosome Maintenance Complex Component; MCL-1—Myeloid cell leukemia; RAB13—Ras-related protein; FAM27E3—Family With Sequence Similarity 27 Member E3; LINC02367—Long Intergenic Non-Protein Coding RNA 2367; PD-L1—Programmed Death Ligand 1; LAG3—Lymphocyte-Activation Gene 3; IDO1—Indoleamine 2,3-dioxygenase 1; HAVCR2—Hepatitis A virus cellular receptor 2; CXCL1—C-X-C motif chemokine ligand 1; RIPK1—Receptor Interacting Serine/Threonine Kinase 1; RIPK3—Receptor Interacting Serine/Threonine Kinase 3; MLKL—Mixed Lineage Kinase Domain-Like Pseudokinase; PDPN—Podoplanin; LAMB3—Laminin Subunit β 3.

## Data Availability

Not applicable.

## Abbreviations

The following abbreviations are used in this manuscript:

*MYOSLID*	Myocardin-Induced Smooth Muscle LncRNA Inducer of Differentiation
CRC	colorectal cancer
HNSCC	head and neck squamous cell carcinoma
OS	osteosarcoma
OSCC	oral cell squamous carcinoma
HIF1A	hypoxia-inducible factor 1-α
RAB13	Ras-Associated Protein 13
RIPK1	Receptor Interacting Serine/Threonine Kinase 1
